# The Correlation of Red Cell Distribution Width With Peripheral Blood Smear: A Study From a Tertiary Care Hospital in Peshawar

**DOI:** 10.7759/cureus.74229

**Published:** 2024-11-22

**Authors:** Sumira Abbas, Mian Mufarih Shah, Mehwash Iftikhar

**Affiliations:** 1 Department of Pathology, Peshawar Medical College, Kuwait Teaching Hospital, Peshawar, PAK; 2 Department of Medicine, Medical Teaching Institute, Hayatabad Medical Complex, Peshawar, PAK

**Keywords:** anemia, hemolytic disorders, microcytic hypochromic anemia, peripheral blood smear (pbs), red cell distribution width (rdw)

## Abstract

Objective: Anemia is a condition characterized by a shortage of red blood cells (RBCs) and hemoglobin (Hb). A peripheral blood smear (PBS) test involves examining a blood sample to identify important abnormalities in the red blood cells, white blood cells (WBC), and platelets. The aim of this study was to correlate the red cell distribution width (RDW) with peripheral blood smear findings in anemic patients presenting to a tertiary care hospital in Peshawar.

Methodology: This cross-sectional study was conducted at Hayatabad Medical Complex, Peshawar, from January 15, 2023, to July 14, 2023. A total of 450 patients aged 18 years and above with confirmed or suspected anemia (Hb of <13 g/dL in men and <12 g/dL in women), hemolytic disorders, or conditions affecting RBC morphology were included. Peripheral blood smear analysis was performed by two independent hematologists to minimize observer bias. Spearman's rank correlation coefficient was applied to determine the correlation between red cell distribution width and the morphological findings on the peripheral blood smear.

Results: Among 450 anemic patients, 232 (51.6%) were women, and 218 (48.4%) were men. The mean age was 25.72 ± 23.23 years. RBC indices showed microcytic RBCs in 270 (60.0%) patients, normocytic RBCs in 157 (34.9%), and macrocytic RBCs in 23 (5.1%). Hypochromic RBCs were found in 301 (66.9%) cases and normochromic in 109 (24.2%). Peripheral blood smear analysis revealed polychromasia in 37 (8.2%) patients and hypochromia in 247 (54.9%). Statistically significant correlations were observed between red cell distribution width and most PBS findings.

Conclusion: Microcytic hypochromic anemia was the most prevalent type on the peripheral blood smear, showing a significant correlation with red cell distribution width. The strong correlation between RDW and anisocytosis suggests the potential utility of RDW as a screening tool in clinical practice.

## Introduction

Hematological tests are used to diagnose conditions affecting the blood and bone marrow, such as anemia, infections, hemophilia, clotting disorders, leukemia, lymphoma, and myeloma. Common tests in this field include the complete blood count (covering red blood cells {RBCs} and white blood cells {WBC}, platelets, hemoglobin {Hb}, hematocrit {HCT}, red cell volume, differential white blood cell count, and other red cell measures), as well as prothrombin and partial thromboplastin times. More specialized procedures, such as bone marrow biopsies and aspirates, are employed for diagnosing blood-related diseases. These conditions and their treatments can have significant health impacts and negatively affect a patient's quality of life [1].

Anemia remains a significant global health issue, particularly in developing nations [2,3]. According to the World Health Organization (WHO), anemia affects 1.62 billion people, representing 24.8% of the global population [4]. Studies show that 40% of children aged 6-59 months, 37% of pregnant women, and 30% of women aged 15-49 years suffer from anemia [5]. Severe anemia is defined as hemoglobin (Hb) levels below 70 g/L in children under five and below 80 g/L in other age groups [6]. Addressing anemia involves targeting its underlying causes, such as nutritional deficiencies, chronic illnesses, or genetic factors, to improve patient outcomes.

Abnormalities in the size, shape, or color of red blood cells (RBCs) such as anisocytosis, poikilocytosis, and hypochromia are important indicators in differentiating between types of anemia. For instance, microcytic and hypochromic RBCs are typically associated with iron deficiency anemia, whereas macrocytic RBCs may indicate malnutrition or deficiencies in vitamin B12 or folate [7].

Anemia is diagnosed through a peripheral blood smear (PBS), where microscopic examination provides insight into variations in the shape and size of RBCs or the presence of inclusion bodies. This analysis can reveal abnormalities in RBC size and shape, aiding in the identification of anemia [8]. Anemia can also be categorized morphologically using RBC indices, which are derived from RBC and hemoglobin analyses. These indices help differentiate between microcytic, macrocytic, and normocytic anemias by measuring the size and Hb concentration in RBCs [9]. For instance, the mean corpuscular volume (MCV) reflects the average volume of RBCs in femtoliters (fL), typically ranging from 80 to 100 fL. The mean corpuscular hemoglobin (MCH) measures the average weight of Hb per cell, usually between 27 and 33 pg. The mean corpuscular hemoglobin concentration (MCHC) represents the average Hb weight relative to RBC volume, typically falling between 32 and 36 g/dL. Abnormal results in these indices influence the diagnosis and classification of anemia [10].

When the average size of RBCs is smaller than normal, it is referred to as microcytic anemia (MCV < 80 fL), often seen in chronic iron deficiency anemia. Macrocytic anemia, characterized by an abnormally large mean RBC volume (MCV > 100 fL), can result from conditions such as vitamin B12 deficiency. Normocytic anemia occurs when there is a reduced RBC count, but the MCV remains within the typical range of 80-100 fL. This type of anemia can be caused by factors such as hemolytic anemia, nutritional deficiencies, or kidney failure [11].

An automated hematology analyzer generates a graphical representation called an RBC histogram, which is a routine part of a complete blood count. This tool aids in diagnosing various RBC disorders and provides critical data on RBC parameters, such as red cell distribution width (RDW), MCH, and MCV [12]. The histogram plots cell size on the x-axis and the total number of cells on the y-axis, offering vital insights for diagnosing and managing severe RBC-related conditions [13].

Interpreting PBS morphology is a fundamental skill for hematologists and is crucial for providing timely diagnoses. Morphological evaluations can uncover pathological findings that enable prompt diagnosis and swift treatment of conditions such as intracellular parasitic infections, hemolytic anemias, suspected thrombotic microangiopathies (TMA), and acute leukemias [14,15].

The correlation of RBC indices and morphology with peripheral blood smear is essential in diagnosing and managing various hematological disorders. RBC indices provide quantitative data about red blood cell size, hemoglobin content, and concentration. Conversely, the morphology observed in peripheral blood smears offers qualitative insights, such as cell shape, size variations, or the existence of abnormal cells, which may not always be captured by indices alone. In clinical practice, combining these two approaches enhances diagnostic accuracy, particularly in cases of anemia, thalassemia, and other blood disorders. This study aims to correlate the red cell distribution width with peripheral blood smear findings to evaluate its potential as a reliable screening tool in clinical practice.

## Materials and methods

This cross-sectional study was conducted at Hayatabad Medical Complex, Peshawar, from January 15, 2023, to July 14, 2023. The ethical approval was obtained from the Medical Teaching Institute (MTI)-Hayatabad Medical Complex Hospital Research and Ethical Committee (IREB) (approval number: HMC/ERC/2023/2286). Using a non-probability consecutive sampling technique, a total of 450 patients were enrolled in the study. The study included patients of all genders, aged 18 years and above, with confirmed or suspected anemia (Hb of <13 g/dL in men and <12 g/dL in women), hemolytic disorders, or other conditions affecting RBC morphology, who had clinical indication for PBS analysis and RBC index assessment. Patients under 18 years of age, those with a history of blood transfusions within the previous three months, known hematological malignancies, and current medications affecting RBC morphology; and individuals unable to provide informed consent were excluded from the study.

Demographic details and medical history including previous anemia diagnoses, chronic diseases, current medications, and comorbidities were documented after obtaining informed consent from each patient. Blood samples were collected through standardized venipuncture methods following WHO guidelines. Complete blood counts were conducted using a Sysmex XN-1000 Automated Hematology Analyzer (Kobe, Japan) to measure RBC indices, including MCV, MCH, and MCHC. Peripheral blood smears were prepared using the wedge technique and stained with Giemsa stain for the morphological analysis of RBCs. Two independent hematologists examined the smears under a microscope to minimize observer bias, with each being blinded to the other's findings and the automated analyzer results. In cases of discrepancy, a third senior hematologist's opinion was sought.

The data was analyzed using SPSS version 23.0 (IBM Corp., Armonk, NY). Descriptive statistics for age, gender, and RBC indices were documented as frequencies, percentages, means, and standard deviations as appropriate. The choice of Spearman's rank correlation coefficient was based on the non-normal distribution of data (confirmed by the Shapiro-Wilk test) and the presence of ordinal variables. This test was applied to determine the correlation between red cell distribution width and the morphological findings on the peripheral blood smear. A p-value of <0.05 was considered statistically significant.

## Results

The study included 450 anemic patients, with a slight female predominance (232, 51.6%) compared to men (218, 48.4%). The mean age of the patients was 25.72 ± 23.23 years. Regarding hematological parameters, the mean WBC count was 12.04 ± 23.72 × 10⁹/L, and the mean RBC count was 5.49 ± 24.68 × 10¹²/L. Hemoglobin levels averaged 10.29 ± 5.85 g/dL, while the hematocrit (HCT) was 31.02% ± 8.65%. The red cell indices demonstrated a mean MCV of 76.65 ± 13.01 fL, a mean MCH of 24.61 ± 5.51 pg, and a mean MCHC of 31.93 ± 2.97 g/dL. The red cell distribution width-coefficient of variation (RDW-CV) was 17.28% ± 8.21%. Regarding platelet counts, the mean platelet value was 742.02 ± 7314.02 × 10⁹/L. The differential showed neutrophils of 58.20% ± 20.11%, lymphocytes of 31.72% ± 18.37%, monocytes of 6.35% ± 3.25%, eosinophils of 2.41% ± 3.32%, and basophils of 0.05% ± 0.54%. Metamyelocytes were 0.07% ± 0.57%, myelocytes were 0.52% ± 3.06%, and blast cells were 0.17% ± 3.58%, while promyelocytes were absent. Reticulocytes were 1.56% ± 2.75% (Table 1).

**Table 1:** Red cell indices and complete blood count in patients with anemia (n = 450). The data is presented as N, %, and mean ± SD. SD: standard deviation

Variable		Mean ± SD, n (%)
Gender	Male	218 (48.4%)
	Female	232 (51.6%)
Age (years)		25.72 ± 23.23
White blood cells (10^9^/L)		12.04 ± 23.72
Red blood cells (10^12^/L)		5.49 ± 24.68
Hemoglobin (g/dL)		10.29 ± 5.85
Hematocrit (HCT)		31.02 ± 8.65
Mean corpuscular volume (MCV) (fL)		76.65 ± 13.01
Mean corpuscular hemoglobin (MCH) (pg)		24.61 ± 5.51
Mean corpuscular hemoglobin concentration (MCHC) (g/dL)		31.93 ± 2.97
Red cell distribution width-coefficient of variation (RDW-CV) (%)		17.28 ± 8.21
Platelets (10^9^/L)		742.02 ± 7314.02
Neutrophils (%)		58.20 ± 20.11
Lymphocytes (%)		31.72 ± 18.37
Monocytes (%)		6.35 ± 3.25
Eosinophils (%)		2.41 ± 3.32
Basophils (%)		0.05 ± 0.54
Metamyelocytes (%)		0.07 ± 0.57
Myelocyte (%)		0.52 ± 3.06
Promyelocytes (%)		0.00 ± 0.00
Blast cells (%)		0.17 ± 3.58
Reticulocytes (%)		1.56 ± 2.75

In the analysis of RBC indices, 270 (60.0%) patients had microcytic RBCs, while 157 (34.9%) had normocytic RBCs, and 23 (5.1%) had macrocytic RBCs. For hemoglobin content, 301 (66.9%) were classified as hypochromic, and 109 (24.2%) were normochromic. Additionally, 38 (8.4%) patients had pencil cells, 45 (10%) had teardrop cells, and only two (0.4%) showed rouleaux formation. Most individuals, 364 (80.9%), showed no significant morphological abnormalities. Basophilic stippling was observed in five (1.1%) cases, while a dimorphic blood picture was observed in one (0.2%) (Table 2).

**Table 2:** Red blood cell (RBC) morphology and hemoglobin characteristics. The data is presented as N and %.

Variable		n	%
Red cell size	Microcytic (<80 fL)	270	60.0
	Normocytic (80-97 fL)	157	34.9
	Macrocytic (>97 fL)	23	5.1
Hemoglobin in RBC	Hypochromic (<27 pg)	301	66.9
	Normochromic (27-31 pg)	109	24.2
	High hemoglobin (>31 pg)	40	8.9
Morphology	Pencil cells	38	8.4
	Teardrop cells	45	10.0
	Rouleaux formation	2	0.4
	Contract cells	1	0.2
	Nil	364	80.9
Basophilic stippling	Yes	5	1.1
	No	445	98.9
Dimorphic blood picture (both microcytic and macrocytic)	Yes	1	0.2
	No	449	99.8

The peripheral blood smear analysis revealed polychromasia in 37 (8.2%) patients and hypochromia in 247 (54.9%). Anisocytosis was noted in 212 (47.1%) samples, macrocytosis in 206 (45.8%) cases, and microcytosis in 374 (83.1%) patients. Normocytic RBCs were found in two (0.4%) individuals. Regarding RBC shape, elliptocytes were present in 23 (5.1%) cases, poikilocytosis in 107 (23.8%), and both acanthocytes and echinocytes in three (0.7%) each. The fragmentation of RBCs was noted in 14 (3.1%), rouleaux formation in 20 (4.4%), RBC agglutination in two (0.4%), and nucleated RBCs in 33 (7.3%) cases (Table 3).

**Table 3:** Common RBC microscopic findings in peripheral blood smear. The data is presented as N and %. RBC: red blood cell

Variable			n	%
Color of RBCs	Polychromasia	Yes	37	8.2
		No	413	91.8
	Hypochromia	Yes	247	54.9
		No	203	45.1
	Normochromic	Yes	2	0.4
		No	448	99.6
Size of RBCs	Anisocytosis	Yes	212	47.1
		No	238	52.9
	Macrocytosis	Yes	206	45.8
		No	244	54.2
	Microcytosis	Yes	374	83.1
		No	76	16.9
	Normocytic	Yes	2	0.4
		No	448	99.6
Shape of RBCs	Elliptocytes	Yes	23	5.1
		No	427	94.9
	Poikilocytosis (teardrop cells)	Yes	107	23.8
		No	343	76.2
	Acanthocytes	Yes	3	0.7
		No	447	99.3
	Echinocytes (crenated RBC)	Yes	3	0.7
		No	447	99.3
Distribution of RBCs	Fragmentation	Yes	14	3.1
		No	436	96.9
	Rouleaux formation	Yes	20	4.4
		No	430	95.6
	RBC agglutination	Yes	2	0.4
		No	448	99.6
	Nucleated RBCs	Yes	33	7.3
		No	417	92.7

The correlation analysis between red cell distribution width and peripheral smear findings revealed that RBC morphology demonstrated a moderate positive significant correlation (ρ = 0.383; p < 0.001). Spherocytes showed a weak negative significant correlation (ρ = -0.133; p = 0.005), while RBC agglutination showed a weak negative significant correlation (ρ = -0.096; p = 0.043). Nucleated RBCs showed a moderate negative significant correlation (ρ = -0.266; p < 0.001), and polychromasia had a weak negative significant correlation (ρ = -0.168; p < 0.001). Hypochromia, poikilocytosis, and anisocytosis demonstrated moderate negative significant correlations (ρ = -0.407, -0.414, and -0.592, respectively; p < 0.001). Macrocytosis showed a weak positive significant correlation (ρ = 0.241; p < 0.001), while microcytosis showed a weak positive significant correlation (ρ = 0.133; p = 0.005). Elliptocytes demonstrated a weak negative but significant correlation (ρ = -0.144; p = 0.002) (Table 4).

**Table 4:** The correlation of peripheral blood smears with the red cell distribution width. *P < 0.05 is considered significant. **P < 0.001 is considered highly significant. RBC: red blood cell

Variable	Red cell distribution width	
	ρ	P-value
Morphology of RBCs	0.383**	<0.001
Basophilic stippling	-0.055	0.245
Spherocytes	-0.133**	0.005
Rouleaux formation	-0.027	0.570
Crenated RBC	0.019	0.691
RBC agglutination	-0.096*	0.043
Dimorphic blood picture	0.078	0.100
Nucleated RBCs	-0.266**	<0.001
Polychromasia	-0.168**	<0.001
Fragmentation	-0.151**	0.001
Hypochromia	-0.407	<0.001
Poikilocytosis	-0.414	<0.001
Anisocytosis	-0.592	<0.001
Macrocytosis	0.241	<0.001
Microcytosis	0.133	0.005
Acanthocytes	-0.003	0.954
Normocytic	-0.017	0.722
Elliptocytes	-0.144	0.002
Normochromic	-0.006	0.903

## Discussion

For decades, peripheral blood examination has been a valuable tool for understanding hematological changes. Today, the use of automated hematology analyzers in laboratories is considered both standard practice and a critical necessity [16]. However, peripheral blood smear analysis remains the gold standard for diagnosing a wide range of red blood cell, white blood cell, and platelet disorders [17].

Another prospective comparative study identified the types of anemia through the analysis of PBS, automated RBC histograms, and RBC indices, aiming to compare and correlate the findings from RBC indices and histograms with PBS results. Among the 500 samples, the most common type of anemia was normocytic normochromic anemia, found in 272 cases (54.4%), followed by microcytic hypochromic anemia in 183 cases (36.6%), macrocytic anemia in 27 cases (5.4%), normocytic hypochromic anemia in 10 cases (2%), and dimorphic anemia in eight cases (1.6%) [18]. These results were consistent with previous studies, such as Samly et al., where 61 out of 110 patients (55.4%) had normocytic normochromic anemia [7], and Agarwal et al., where 40 out of 100 patients (40%) had the same condition [19]. The present study was inconsistent with the above-reported studies and indicated that microcytic (374, 83.1%) and hypochromic (247, 54.9%) anemia were the most prevalent types. Macrocytosis was reported in 206 (45.8%) patients.

A comparative cross-sectional study was carried out to compare the results of manual PBS examinations to red cell indices and histograms in anemic patients. Of the 250 anemic samples analyzed, 58.4% were from women and 41.6% from men. Based on hemoglobin levels, 44.4% of cases had severe anemia, followed by 36.8% with moderate anemia. The manual PBS examination revealed that the most common type of anemia was microcytic hypochromic anemia (45.2%), while red cell indices and histograms indicated that normocytic normochromic anemia (48.0%) was the most frequent. There was a statistically significant difference (p < 0.001) between the manual PBS examination and the automated hematological analyzer [20]. These findings align with those from studies by Jain et al. [21], Jansari et al. [22], and Ashok and Varadarajan [23], where microcytic hypochromic anemia was the most common type identified through PBS. However, the results differ from the study by Samly et al., where normocytic normochromic anemia was most prevalent (57.0%) [7]. This difference could be attributed to variations in study regions, as noted by Samly et al. [7].

Similarly, another study observed that normocytic normochromic anemia was the most common type, according to red cell indices obtained from an automated hematological analyzer, occurring in 48.0% of cases [24]. These results were consistent with the study by Samly et al. [7], where normocytic normochromic anemia was also the predominant type, followed by microcytic hypochromic anemia, as identified by the automated analyzer. However, the findings of this research differ from studies by Jain et al. [21], Jansari et al. [22], Garg et al. [25], and Ashok and Varadarajan [23], where microcytic hypochromic anemia was more common, followed by normocytic normochromic anemia, based on red cell indices. In the present study, both RBC indices and peripheral blood smear results indicated that microcytic hypochromic anemia was the most prevalent type.

Similarly, another study correlated peripheral blood smear findings with RBC histogram patterns and RBC indices to enhance the diagnostic approach for anemia. Among the 500 anemic cases, the peripheral smear examination identified 21.8% normocytic normochromic, 46% microcytic hypochromic, 8.8% macrocytic, 17.6% dimorphic, and 5.8% hemolytic anemia. The erythrocyte indices and histogram analysis yielded similar results, except for macrocytic (15.6%), dimorphic (15.8%), and hemolytic (0.8%) anemia. The correlation between the two methods was statistically significant (p < 0.0001) [24]. The present study showed partial similarity to the above research, with RBC indices indicating microcytic (260, 60.0%), normocytic (157, 34.9%), hypochromic (301, 66.9%), normochromic (109, 24.2%), and dimorphic (one, 0.2%) cases. In contrast, peripheral smear analysis revealed microcytosis (374, 83.1%), macrocytosis (206, 45.8%), anisocytosis (212, 47.1%), hypochromia (247, 54.9%), and poikilocytosis (107, 23.8%). A statistically significant correlation was found between peripheral blood smear findings and red cell distribution width (p < 0.001).

Interestingly, another study examined RBC morphology through peripheral blood smear analysis in 350 anemia patients and compared these observations with RBC indices, including MCV, MCHC, and MCH. Of the 350 cases, the majority (171, 48.85%) of patients were adults between 21 and 50 years old. Women made up 227 (64.86%) of the cases, while 123 (35.14%) were men. The most frequent type of anemia identified was microcytic hypochromic anemia, accounting for 210 cases (60%), followed by dimorphic anemia in 73 cases (20.86%) [26]. These results were consistent with studies by Mishra et al. [27] and Jain et al. [21], who also found microcytic and hypochromic anemia to be the most common types, representing 47% and 40% of cases, respectively [27,21]. These findings were corroborated with the present study that showed the mean age of patients was 25.72 ± 23.23 years, reflecting a broad age range among the individuals, with a slight female predominance of 232 (51.6%) compared to men in 218 (48.4%). The most prevalent anemia found on smear analysis was microcytic and hypochromic anemia, which were observed in 374 (83.1%) and 247 (54.9%) cases, respectively. While on red blood cell indices, it was seen in 270 (60%) and 301 (66.9%) cases, respectively.

This study had a few limitations. The use of a non-probability sampling technique may affect the representativeness of the sample. Conducting the study at a single tertiary care hospital in Peshawar may limit the generalizability of the results to other geographic or healthcare settings. Moreover, this study did not correlate the findings of anemia with other biochemical parameters such as serum iron studies in iron deficiency anemia, vitamin B12 studies in megaloblastic anemia, and reticulocyte count. Future studies should consider using a probability sampling technique and multiple centers to improve the representativeness of the sample.

## Conclusions

This study concluded that microcytic hypochromic anemia was the most prevalent type on peripheral blood smear, which correlated well with red cell distribution width. Notably, anisocytosis showed significant correlations with red cell distribution width. The correlation patterns observed between RDW and various morphological features suggest that RDW could serve as a valuable initial screening parameter in clinical practice. These findings indicate the potential utility of RDW as a reliable screening tool that could help optimize the use of peripheral blood smear analysis in resource-limited settings. Future multicenter studies incorporating biochemical parameters would be valuable to further validate these findings and establish comprehensive diagnostic algorithms.
